# Advances in the Relationship Between Regulator of Ribosome Synthesis 1 (RRS1) and Diseases

**DOI:** 10.3389/fcell.2021.620925

**Published:** 2021-02-25

**Authors:** Yanan Hua, Jinlian Song, Cuixiu Peng, Runze Wang, Zhongliang Ma, Jinyu Zhang, Zheng Zhang, Ning Li, Lin Hou

**Affiliations:** ^1^Department of Neurobiology, Basic Medical College, Qingdao University, Qingdao, China; ^2^Department of Laboratory, Women and Children’s Hospital of Qingdao, Qingdao, China; ^3^Department of Biochemistry and Molecular Biology, Basic Medical College, Qingdao University, Qingdao, China; ^4^Department of Breast Surgery, The Affiliated Hospital of Qingdao University, Qingdao University, Qingdao, China

**Keywords:** RRS1, ribosome, disease, cancer, molecular mechanism

## Abstract

A regulator of ribosome synthesis 1 (RRS1) was discovered in yeast and is mainly localized in the nucleolus and endoplasmic reticulum. It regulates ribosomal protein, RNA biosynthesis, and protein secretion and is closely involved in cellular senescence, cell cycle regulation, transcription, translation, oncogenic transformation etc., Mutations in the RRS1 gene are associated with the occurrence and development of Huntington’s disease and cancer, and overexpression of RRS1 promotes tumor growth and metastasis. In this review, the structure, function, and mechanisms of RRS1 in various diseases are discussed.

## Introduction

Ribosomes are the sites of protein synthesis and consist of ribosomal RNA (rRNA) and ribosomal proteins (RPs). In mammalian ribosomes, rRNA and RPs are arranged into large (60S) and small (40S) subunits. The RPs also have functions independent of ribosomes, such as the regulation of DNA repair, cell proliferation, apoptosis, and differentiation (Warner and Mcintosh, 2009). Furthermore, RPs participate in the coordination of protein synthesis in the cytoplasm and mitochondria (Surguchov, 1987). Warner and McIntosh suggested that the extra-ribosomal functions of RPs can be classified as follows: (1) regulation and maintenance of the ribosome assembly, (2) identification of the nucleolar stress responses to mediate G phase cell cycle arrest, and (3) apoptosis and other specific or non-specific functions. Tsuno et al. discovered the regulator of the ribosome synthesis 1 (RRS1) protein in yeast cells. It is located in the nucleolus and endoplasmic reticulum, and is involved in ribosome biogenesis, 25S rRNA maturation, and the assembly of the 60S subunit (Surguchov, 1987). Aberrant expression levels of RRS1 affect ribosome biogenesis, which in turn has pathological consequences.

## The Structure of RRS1 Gene and Its Coding Protein

The human RRS1 gene is 1,714 bp long and is located in chromosome 8q13.1. It contains only one exon (NCBI accession number: NC_000008.11) that is transcribed into a single mRNA transcript without any alternatively spliced isoforms. The 1,079 bp open reading frame (ORF) of RRS1 encodes for the RRS1 protein containing 365 amino acid residues with a relative molecular mass of 41,193 KDa. The RRS1 protein is mainly localized to the nucleolus and endoplasmic reticulum. The C-terminal residues 302–365 are rich in arginine, glycine and lysine (Arg/Gly/Lys). The first methionine residue at the N-terminus can be modified by acetylation, and Arg344 at the C-terminus is the phosphorylation site.

## The Biological Function of RRS1

### RRS1 Is Involved in Ribosome Biosynthesis

Cellular proliferation requires high rates of protein synthesis, which in turn is dependent on ribosome biogenesis (Warner and Mcintosh, 2009). RRS1 regulates 25S rRNA maturation and 60S subunit assembly (Tsuno et al., 2000). Tsujii et al. (2000) found that RRS1 formed a complex with the nucleolus protein EBP2 in yeast cells, which bound to and stabilized the pre-ribosomal subunit and promoted ribosome biosynthesis. RRS1 also binds to the ribosomal protein RPL11 on the 60S subunit, indicating its important role in ribosome biogenesis (Miyoshi et al., 2002). In *Saccharomyces cerevisiae*, RRS1 interacts with RPF2 (ribosome production factor 2) to recruit RPL5 and RPL11, to synthesize the 60S subunit (Fatica and Tollervey, 2002; Fromont-Racine et al., 2003; Granneman and Baserga, 2004). In the absence of RRS1 and RPF2 binding, the immature pre-RNA is exported from the nucleolus to the nuclear mass but not to the cytoplasm, thereby affecting 60S subunit biosynthesis (Zhang et al., 2007). Missense mutations in the C-terminal of RRS1 have been detected in secretion-deficient yeast cells, indicating that it plays an important role in protein synthesis and secretion. Morita et al. showed that Rpf2 binds to both RRS1 and RPL11 in yeast cells and regulates the 27-SB pre-rRNA transformation during 25S and 60S ribosomal subunit biosynthesis (Morita et al., 2002). Miyoshi et al. found that RRS1 interacts with the 60S subunit during biogenesis, maturation, assembly, and export from the nucleus to the cytoplasm (Miyoshi et al., 2004; Nariai et al., 2005). Zhang et al. found that RRS1 and Rpf2 recruit 5S rRNA, RPL5, and RPL11 to the newly produced ribosomes (Zhang et al., 2007). Therefore, the main role of RRS1 in ribosomal biosynthesis is to recruit the necessary factors and to form a stable structure, especially for the assembly and nucleation of 60S subunit.

### RRS1 Regulates Chromosomal and Telomeric Arrangement During the Cell Cycle

The process of mitosis is divided into distinct stages namely interphase, prophase, metaphase, anaphase, and telophase. During the prophase, the chromosomes start to condense and gradually converge to the equatorial plate by a metaphase (Kuriyama and Borisy, 1982). Gambe et al. (2009) found that RRS1 silencing in HeLa cell lines increased the proportion of cells in the early metaphase and disrupted chromosomal arrangement on the equatorial plate, resulting in a delayed anaphase. The accumulation of chromosomes in the equatorial plate depends on the cohesion of sister chromatids, and their attachment to the spindle microtubules. RRS1-knockdown HeLa cells treated with colchicine and MG132 showed premature separation of the sister chromatids and lower levels of the centromere protein Shugoshin 1, resulting in abnormally arranged chromosomes on the equatorial plate (Losada, 2008). Thus, RRS1 controls chromosomal accumulation on the equatorial plate during cell cycle (Gambe et al., 2009).

The telomeres are located at the termini of linear chromosomes, and consist of TTAGGG repeat sequences and related proteins that are protected by a cap structure (Chan and Blackburn, 2002; Palm and De, 2008). SIR4 and yKu are partially bound to the N-terminus of the membrane protein s3 (MPs3) during the S phase of the cell cycle to create a niche for anchoring telomeres (Hediger et al., 2002; Taddei et al., 2004; Bupp et al., 2007; Schober et al., 2009). This process is stabilized by the EBNA1 binding protein 2 (EBP2) and RRS1, which interact with both MPs3 and the C-terminus of Sir4. Yeast cells with mutated EBP2 and RRS1 show aberrant telomere aggregation (Horigome et al., 2011). Therefore, RRS1 and EBP2 also affect telomere aggregation.

### RRS1 Weakens Cell Senescence

Due to a limitation of the number of proliferating cycles, the inactivation of oncogenes, activation of tumor suppressor genes, drugs that induce DNA damage, and cell senescence affection, most mammalian cells cannot proliferate indefinitely (Campisi and d’Adda di Fagagna, 2007; Kuilman et al., 2010; Pérezmancera et al., 2014). Although induction of senescence in cancer cells can inhibit tumor growth or elicit an immune response to clear precancerous cells, recent studies show that senescent cells lead to age-related dysfunction through inflammatory reactions (Campisi and d’Adda di Fagagna, 2007; Xue et al., 2007; Baker et al., 2011; Kang et al., 2015). Telomerase dysfunction, DNA damage response, and oxidative stress induce senescence *via* the p53-p14/p19Arf and p16INK4A-RB pathways (Schramek et al., 2011). The tumor suppressor p53 is normally expressed at low levels due to its interaction with the E3 ubiquitin ligase murine double minute 2 (MDM2) (Kuilman et al., 2010; Lee and Gu, 2010). It is activated in response to cellular stresses with the detachment of MDM2, and transcriptionally activates downstream targets effectors of cell cycle arrest, apoptosis, DNA repair, and cell senescence (Levine and Oren, 2009). Recent studies show that the nucleoli can sense various stresses and activate the p53 pathway (Lee and Gu, 2010). Following nuclear stress, several ribosomal proteins including nucleolar phosphate protein, nucleolar protein, nuclear factor, RL11, RPL5, RPL23, and RPS7 are released. These proteins directly bind to MDM2 to prevent ubiquitin-mediated degradation of p53, thereby maintaining normal cell proliferation (Boulon et al., 2010). Bhat’s study showed that RPL11 is involved in the p53 nuclear stress response pathway (Manfredi, 2010). Increased RPL11 translocation is observed in cells with aberrant 40S ribosomal subunit biogenesis, and RPL11 translation promotes p53 activation (Bhat et al., 2004). Some studies show that RPL11 regulates MDM2 as part of the 5S ribonucleoprotein (RNP) complex consisting of RPL11, RPL5, and 5S rRNA (Wilson et al., 1996; Sugawara et al., 1999; Fumagalli et al., 2012; Donati et al., 2013). Miyoshi et al. found that RRS1 binds to RPL11, and enhances the binding of p53 to MDM2, which downregulates p53 activity (Miyoshi et al., 2002). Thus, overexpression of RRS1 diminishes p53-mediated cellular senescence.

## The Relationship Between RRS1 and Diseases

### The Relationship Between RRS1 and Huntington Disease

Huntington disease (HD) is a neurodegenerative disease characterized by involuntary limb movement and progressive dementia. Endoplasmic reticulum stress (ERS) has recently been identified as the molecular basis of HD pathogenesis (Duennwald and Lindquist, 2008; Reijonen et al., 2008). The ER is extremely sensitive to cellular stresses such as low energy levels, abnormal redox state, and high calcium influx, and can trigger apoptosis. Therefore, ERS is closely related to the genesis and progression of various diseases (Harding and Ron, 2002; Mouw et al., 2003). RRS1 mRNA levels were significantly increased in the brain tissues of a HD (Hdh^+/–^ and Hdh ^–/–^) mouse model, as well as in the brain tissues of HD patients relative to age-matched healthy controls (Fossale et al., 2002). Furthermore, RRS1 and metadherin are co-localized on the ER in HD mice. RRS1 is activated during ERS and relays the signals to metadherin. Therefore, RRS1 plays an important role in the pathogenesis of HD by promoting ERS (Carnemolla et al., 2009).

### The Role of RRS1 and Cancer

Abnormal ribosome biosynthesis can trigger the ribosomal stress response, which activates the oncogenic p53-HDM2 feedback pathway (Macias et al., 2010; Montanaro et al., 2012). Aberrant expression of RPL5, RPL11, RPL23, and RPS7 can also promote the ribosomal stress response and p53-HDM2 feedback pathway (Lohrum et al., 2003; Dai and Lu, 2004; Jin et al., 2004; Chen et al., 2007). Several RPs are aberrantly expressed in human tumors. For instance, RPL29 is significantly up-regulated in colorectal cancer (Wang et al., 1999), and RPS8, RPL12, RPL23A, RPL27, and RPL30 show high expression levels in diverse cancers. In addition, overexpression of RPS3A promotes tumor formation in nude mice (Naora et al., 1998).

RRS1 is distributed in the periphery of the nucleus in HeLa cells unlike the nuclear localization seen in normal cells. RRS1 silencing delays cell cycle progression in HeLa cells (Gambe et al., 2009). In addition, RRS1 regulates the balance between cytoplasmic membrane and endoplasmic reticulum, and its dysregulation in tumor cells can significantly alter their physiological functions.

#### The Relationship Between RRS1 and Cervical Cancer

Cervical cancer is one of the leading causes of death among women worldwide (Jemal et al., 2011), and its incidence and fatality rates has increased significantly in developing countries. It ranks second after breast cancer as the most common cancer in females (Li et al., 2013). Although the exact pathological mechanism is unknown, virus infection and genetic mutations have been implicated in the development of cervical cancer. Gambe et al. (2009) detected high levels of RRS1 in the nuclear periphery of cervical cancer Hela cells, which bound to other nucleolar proteins during mitosis and interphase. The expression level of RRS1 was particularly increased at prophase during nucleolus breakage. RRS1 silencing significantly increased the number of tetraploid cells and significantly prolonged cell division, indicating a role in chromosome segregation (Gambe et al., 2009). Finally, down-regulation of RRS1 by miRN-148a inhibited the proliferation, invasion, and migration of cervical cancer cells (Zhang et al., 2019). Therefore, RRS1 promotes cervical cancer development and progression.

#### The Relationship Between RRS1 and HCC

Hepatocellular carcinoma is a lethal malignancy and ranks third in terms of mortality rates after gastric cancer and esophageal cancer (Zimber and GesPaeh, 2008). The incidence of liver cancer has increased significantly in recent years, and patient prognosis is dismal due to a < 10% 5 year survival rate. RRS1 is overexpressed in liver cancer tissues relative to the para-carcinoma tissues, and the overall and disease-free survival rates of patients with high RRS1 expression are significantly lower than that in RRS1low patients. Wang et al. found that RRS1 gene silencing in the liver cancer cell line SMMC7721 induced G1 phase arrest, which effectively blocked cell division and proliferation (Wang et al., 2017). In addition, RRS1 promoted HCC cell proliferation and colony formation, and inhibited apoptosis (Wang et al., 2017). Therefore, RRS1 functions as a pro-tumorigenic factor in liver cancer.

#### The Relationship Between RRS1 and Breast Cancer

**FIGURE 1:**
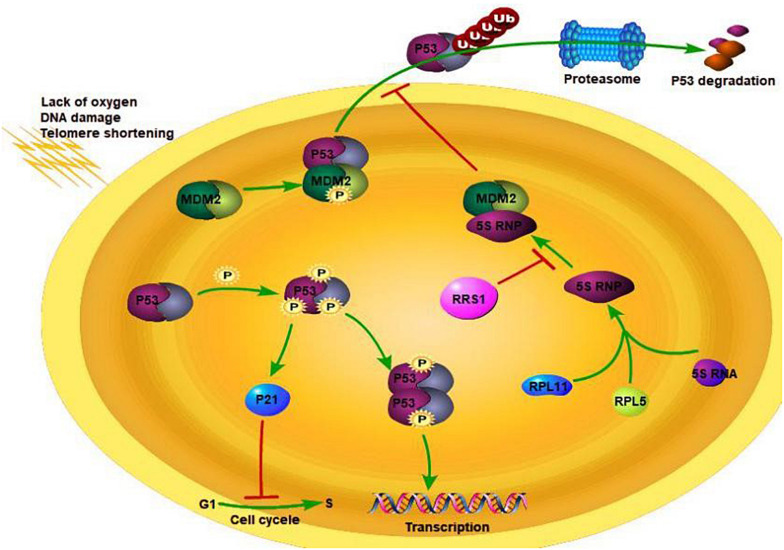
RSS1 regulation mechanism. Adapted from Cao (2019).

Breast cancer is a common malignancy among females, and its incidence rate is increasing every year. It exhibits high genetic heterogeneity (Miller et al., 2016; Turashvili and Brogi, 2017), and targeted therapies against specific gene expression patterns have significantly improved the survival rate of breast cancer patients (Staaf et al., 2010). We found that the RRS1 gene is highly expressed in breast cancer cells, and its knockdown significantly reduced the proliferation rate and increased apoptosis (Hua et al., 2019). RRS1 may promote proliferation of breast cancer through p53 activation via RPL11/MDM2 (Figure 1) (Song et al., 2018). Taken together, RRS1 is a novel oncogene of breast cancer and a promising therapeutic target.

#### The Relationship Between RRS1 and PTC

Papillary thyroid carcinoma (PTC) is one of the most common malignancies of the endocrine system, though the mechanisms underlying the pathogenesis, progression, and prognosis are still largely unclear (Burman and Wartofsky, 2015). Chen et al. recently found that RRS1 silencing in PTC cells inhibited their proliferation by inducing G2/M phase arrest and promoting apoptosis. In addition, RRS1 knockdown promoted metabolism and inhibited vascular development in the *in vivo* PTC model (Chen et al., 2018). Thus, RRS1 is a potential oncogene in PTC and should be further explored.

#### The Relationship Between RRS1 and Colorectal Cancer

Colorectal cancer (CRC) is the third most common malignancy and the fourth leading cause of cancer-related deaths globally (Alwan, 2007; Siegel et al., 2012). Several mutations have been identified as risk factors for the occurrence and progression of CRC (Fearon, 2011; Vermeulen et al., 2013), and their biological relevance have been validated in functional *in vitro* and *in vivo* studies (Hussain et al., 2000; von Karstedt et al., 2015). Therapies targeting proto-oncogenes or tumor suppressors have been developed but their efficacy is limited (Amado et al., 2008; Karapetis et al., 2008; Grothey et al., 2013). Therefore, there is an urgent need to explore new drug targets to treat CRC. Wu et al. found that RRS1 is overexpressed in CRC tissues compared to that in normal tissues and knocking down RRS1 in CRC cells inhibited their proliferation and tumorigenesis in nude mice (Wu et al., 2017). Thus, RRS1 is a potential therapeutic target for CRC.

#### The Relationship Between RRS1 and Gastric Cancer

Gastric cancer (GC) is one of the most common malignant tumors in the digestive system (Song et al., 2017), and the second leading cause of cancer-related deaths worldwide (Karimi et al., 2014). Due to the metastatic potential, recurrence, and chemoresistance of gastric cancer cells, the prognosis of patients with advanced gastric cancer is poor and the overall survival rate is dismal (Fujita, 2009). Studies show that cancer stem cells (CSCs) are the major determinant of gastric tumor metastasis and invasion (Yu et al., 2012). Ma et al. (2019) recently found that miRNA-598 regulates the growth of gastric CSCs by regulating the expression of RRS1.

## Conclusion

RRS1 plays an important role in ribosome biosynthesis, chromosome aggregation in the equatorial plate, and telomere aggregation during cell cycle. In addition, it regulates the cell cycle, ERS, and ribosomal stress response, which affects the p53 signaling pathway. The aberrant expression of RRS1 is associated with Huntington’s disease and cancer development. RRS1 is a potential oncogene in various cancers, and therefore a promising therapeutic target.

## Author Contributions

YH: conceptualization, writing of original draft, project administration, and editing. JS and CP: data curation, formal analysis, funding acquisition, and editing. RW, ZM, JZ, ZZ, and NL: data curation, validation, and editing. LH: conceptualization, supervision, and editing. All authors contributed to the article and approved the submitted version.

## Conflict of Interest

The authors declare that the research was conducted in the absence of any commercial or financial relationships that could be construed as a potential conflict of interest.
